# The “Guidewire-Coil”-Technique to prevent retrograde stone migration of ureteric calculi during intracorporeal lithothripsy

**DOI:** 10.1186/s12894-016-0197-8

**Published:** 2017-01-05

**Authors:** Nici Markus Dreger, Friedrich Carl von Rundstedt, Stephan Roth, Alexander Sascha Brandt, Stephan Degener

**Affiliations:** 1 Department of Urology, Helios Medical Center Wuppertal, Helios University Hospital Wuppertal, University of Witten/Herdecke, Heusnerstraße 40, Wuppertal, 42283 Germany; 2 Scott Department of Urology, Baylor College of Medicine Medical Center, 7200 Cambridge, Houston, TX USA; 3 Department of Urology, Jena Medical Center, Friedrich-Schiller University, Bachstraße 18, Jena, 07743 Germany

**Keywords:** Stone migration, Stone retropulsion, Intracorporeal lithotripsy, Ureterorenoscopy, Ureteric calculi

## Abstract

**Background:**

Stone retropulsion represents a challenge for intracorporeal lithotripsy of ureteral calculi. The consequences are an increased duration and cost of surgery as well as decreased stone-free rates. The use of additional tools to prevent proximal stone migration entails further costs and risks for ureteral injuries. We present the simple technique of using a coil of the routinely used guidewire to prevent stone retropulsion.

**Methods:**

We retrospectively evaluated all patients with mid-to-proximal ureteral stones in 2014, which were treated by ureteroscopic lithotripsy (Ho: YAG and/or pneumatic lithotripsy). The preoperative stone burden was routinely assessed using low dose CT scan (if available) and/or intravenous pyelogram.

**Results:**

The study population consisted of 55 patients with 61 mid-to-proximal calculi. Twentyseven patients underwent semirigid ureterorenoscopy using the “Guidewire-Coil-Technique”, the second group (*n =* 28) served as control group using the guidewire as usual. There has been a statistically significant reduction of accidental stone retropulsion (2/27 vs. 8/28, *p <* 0.05) as well as a decreased use of auxiliary procedures (*p <* 0.05) compared to the control group. No difference was observed in operative time. One ureteral injury in the control group required a prolonged ureteral stenting.

**Conclusion:**

The “Guidewire-Coil-Technique” is a simple and safe procedure that may help to prevent proximal calculus migration and therefore may increase stone-free rates without causing additional costs.

## Background

During the past two decades, there have been many improvements regarding the endoscopic treatment of urolithiasis. Ureterorenoscopy (URS) with and without lithotripsy is a standard method to treat ureteral calculi depending on different factors including location, stone size, individual patient factors as well as equipping [1, 2]. A particular challenge limiting the success of ureteroscopic lithotripsy is stone retropulsion due to insertion of the ureteroscope, pressure by the irrigation fluid and/or the lithotripsy itself [2]. Stone migration occurs in 28–60% of proximal calculi [3–6]. Hereby an increase in operative time, a decrease in stone-free rates and the need for further auxiliary procedures (i.e. shockwave lithotripsy (SWL), flexible ureterorenoscopy (fURS)) with affiliated morbidities and health-care costs have been reported [2, 7, 8]. Novel stone retrieval devices have been introduced to address the problem of accidental stone migration: Stone baskets [9, 10], suction devices [11], balloon catheters [12, 13] guidewire [14–16] and gel-based devices [17, 18] significantly reduced the incidence of stone retropulsion. On the contrary, these devices are associated with additional costs and some of them with a higher risk for ureteral injuries [2].

Because of this predicament, we assessed a new technique only using the usually recommended guidewire to prevent proximal stone migration. We here describe our experience and the efficacy of this method.

## Methods

From January 2014 to December 2014, 55 patients with upper ureteral calculi (*n =* 61) were treated in our institution by primary intracorporeal lithotripsy according to the current guidelines [19]. Preoperative stone location and size were confirmed by abdomen and pelvis computed tomography (CT) scan or in rare cases by intravenous pyelography (IVP), if CT scan was not available. All patients underwent semirigid ureterorenoscopy and intracorporeal lithotripsy has been performed using holmium-YAG laser (Ho:YAG) and/or pneumatic lithotriptor. The “Guidewire-Coil-Technique” in this study was performed by a single faculty urologist (S.R.) with more than 2000 ureterorenoscopies. IRB approval was obtained (no. 43/2016, Witten/Herdecke University).

All 55 patients were analyzed retrospectively: Of these patients 27 were treated using the “Guidewire-Coil-Technique” and 28 patients served as control group using the guidewire in regular fashion. Plain film radiographs of the kidneys, ureters, and bladder (KUB) and sonography were obtained to verify stone-free rate and migration rate.

Patients were stratified by the kind of use of the guidewire. The primary endpoint was the stone-free rate. The incidence of stone retropulsion, need for further auxiliary procedures, operative time and complication rate were defined as secondary endpoints. Statistical assessment was performed using Fisher’s exact test for categorical variables and Mann–Whitney *U* test for continuous variables respectively. *P* values < 0.05 were considered significant. Statistical analysis was performed using SPSS 21® for Mac® (SPSS Inc., Chicago, IL, USA).

### The “Guidewire-Coil-Technique”

**Fig. 1:**
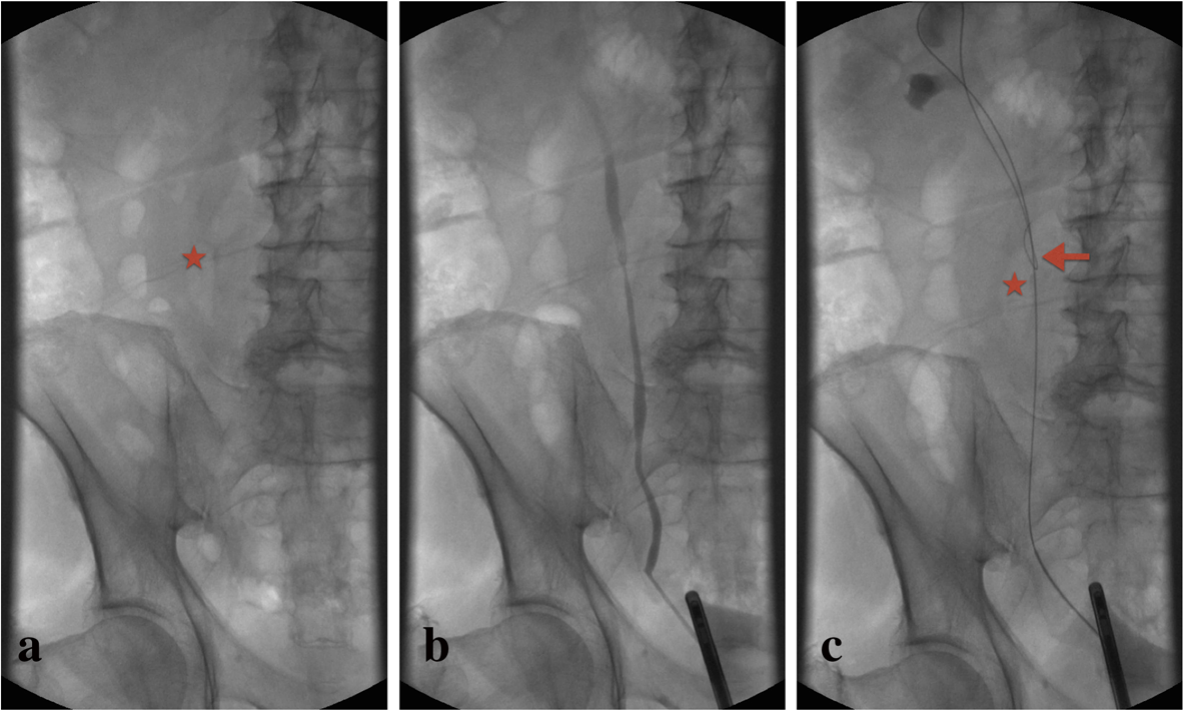
Step-by-step description of the “Guidewire-Coil-Technique” based on the example of a mid ureteric stone. **a**: Plain x-ray; **b**: Retrograde pyelography; **c**: Correctly placed coil of the guidewire. *Asterisk = Ureteral calculus, arrow = Reverted guidewire acting as a counterfort*

**Fig. 2:**
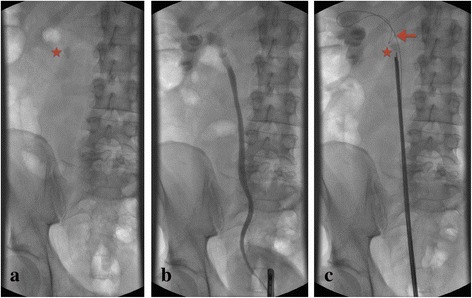
Step-by-step description of the “Guidewire-Coil-Technique” based on the example of a proximal stone. **a**: Plain x-ray; **b**: Retrograde pyelography; **c**: Correctly placed coil of the guidewire. *Asterisk = Ureteral calculus, arrow = Reverted guidewire acting as a counterfort*

**Fig. 3:**
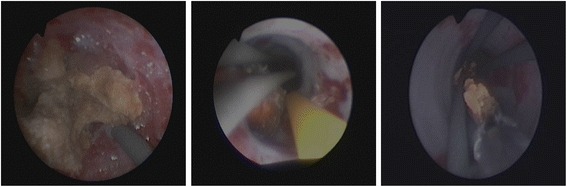
Examples of the endoscopic point of view while using the “Guidewire-Coil-Technique”

In all patients a hydrophilic guidewire with nitinol core and angled tip (outer diameter 0.89 mm (0.035”), length 150 cm, flexible length 30 mm) was used. In our case series, we used a 9.8 F (8 F tip, 9.8 F base) semirigid ureteroscope (Karl Storz, Germany) with a 5 F working channel. After careful retrograde pyelography (RPG, Figs. 1a-b, 2a-b), the guidewire is advanced beyond the stone. After reaching the renal pelvis, the angled tip was placed in the upper calix and then pushed until a loop of the guidewire was achieved. The loop in the upper calix facilitates a direct turn back into the ureter (and prevents a coiling in renal pelvis without turn back in the ureter). By rotating the guidewire manually or with a Halstead clamp (can be helpful with clammy gloves), the angled tip can be used to navigate the guidewire in the desired direction. Additionally, we don’t recommend a guidewire with a straight distal curve because it’s stiffness makes a precise loop in the upper calix much more difficult. The reverted guidewire was placed consecutively directly proximal to the stone (Fig. 1c). At this position, the reverted guidewire acts as a counterfort, which will help to prevent retrograde stone migration during intracorporeal lithotripsy (Figs. 2c and 3).

## Results

The two groups were comparable with regard to gender, age, size or location (Table 1). Upward stone or fragment retropulsion to the kidney occurred in two patients (7.4%) in the treatment and eight patients (28.6%) in the control group, a statistically significant difference (*p <* 0.05, Table 2). In the treatment group a guidewire coiling could be achieved in every patient after 1–8 attempts (median 3 attempts). There was no relevant difference concerning the mean operative time (67.6 versus 70.3 minutes, *p =* 0.901) and the type of lithotripsy used for fragmentation of the stones (Ho:YAG versus pneumatic lithotripsy, *p =* 0.500). Auxiliary procedures such as flexible ureterorenoscopy were necessary in three patients (11%) in the treatment group compared to ten patients (35.7%) in the control group (*p <* 0.05). Postoperatively, patients were followed up with KUB and sonography as described before. The stone-free rates were 92.6% in the treatment and 75% in the control group, respectively. The difference between the two groups was statistically borderline significant (*p =* 0.079). Only one notable (≥ III, classified according to the Clavien system) complication was observed: one patient (3.6%) in the control group had a ureteral wall injury, which resulted in a prolonged ureteral stenting.

**Table 1:** Preoperative characteristics of both groups

	Guidewire-Coil *n =* 27		Control *n =* 28		*P* value
		±SD		±SD	
Gender					0.527^†^
Male	22		22		
Female	5		6		
Age [a]					
Mean	58.0	16.0	55.0	15.5	0.622*
Median	54.0		56.5		
Number of stones	28		33		
Size [mm]					
Mean	9.8	3.4	10.0	3.5	0.953*
Median	8.6		9.2		
Calculus location					
Proximal ureter	13		15		0.571^†^
Mid ureter	15		18		

*SD =* standard deviation

*Significant at *p* value < 0.05 by Mann–Whitney test

^†^Significant at *p* value < 0.05 by Fisher’s-exact test

**Table 2:** Postoperative comparison of both groups

	Guidewire-Coil *n =* 27		Control *n =* 28		*P* value
		±SD		±SD	
Operative time [min]					
Mean	67.6	29.8	70.3	34.0	0.901*
Median	61.0		58.5		
Stone migration					0.044^†^
Yes	2 [7.4%]		8 [28.6%]		
No	25		20		
Auxiliary procedures					0.032^†^
Yes	3 [11%]		10 [35.7%]		
Flexible URS	2		5		
SWL	0		4		
Secondary URS	0		1		
No	24		18		
Stone-free rate					0.079^†^
Yes	25 [92.6%]		21 [75%]		
No	2		7		
Lithotripsy					0.500^†^
Ho:YAG	20		19		
Pneumatic	8		9		

Operative time was determined using the anesthesia protocols

*SD =* standard deviation; URS = ureterorenoscopy; Ho:YAG = Holmium-YAG laser lithotripter

*Significant at *p* value < 0.05 by Mann–Whitney test

^†^Significant at *p* value < 0.05 by Fisher’s-exact test

## Discussion

Stones larger than 5 mm in diameter require intracorporeal fragmentation before extraction through the ureteroscope [20]. A wide variety of endoscopic lithotriptors have become available for stone fragmentation including laser, electrohydraulic and the pneumatic lithotriptor. The ballistic nature of the energy occasionally displaces calculi towards the kidney. Stone migration into the collecting system makes stone retrieval substantially more challenging especially into a lower pole or anterior calyx, which necessitates additional procedures such as adjuvant extracorporeal SWL [8, 21].

Novel stone retrieval devices have been recommended for the prevention of retrograde stone displacement including ureteral stone baskets, balloon catheters, stone cone, etc. (Table 3). However, all of these add to the costs and some increase the risk for ureteral injuries [2].

**Table 3:** Overview of different devices and techniques to prevent accidental stone migration

Author	Year	Device/Technique	*n*	Stone migration [%]	SFR [%]
Kesler et al. [10]	2008	Stone basket (Escape®)	23	n.a.	87
Eisner et al. [22]	2009	Guidewire (Stone Cone®)	133	1.5	98.5
Sen et al. [23]	2014	Guidewire (Stone Cone®)	25	4.5	95.5
Sen et al. [23]	2014	Guidewire (PercSys®)	25	8.7	91.3
Wang et al. [24]	2011	Guidewire (NTrap®)	56	0.0	100
Sen et al. [23]	2014	Gel-based (Lidocaine jelly)	25	21.7	82.6
Mohseni et al. [18]	2006	Gel-based (Lidocaine jelly)	16	12.4	93.7
Rane et al. [25]	2010	Thermosensitive polymer (BackStop®)	34	8.8	87.8
Dretler et al. [13]	2000	Balloon catheter (Passport®)	29	10.3	89.7

The stone-free rate in the current work was different between the 2 groups (92.6% for the treatment group and 75% for the control group). The control group consequently had a higher rate of ancillary procedures as reflected by the significantly different efficiency quotient. This was partly due to stone retropulsion requiring an auxiliary procedure. In comparison to the before mentioned (expensive) stone retrieval devices and their associated stone-free rates (Table 3), our technique was not inferior.

In two patients in the group managed with the guidewire-coil-technique we were not able to prevent stone migration towards the kidney. While we did not observe any proximal stone migration during the placement of the wire there may be an association with the diameter of the dilated ureter (similar to balloon catheters) [2, 13]. Although possible in every patient in the treatment group, it took 1–8 (median 3) attempts to coil the guidewire in the renal pelvis and get a loop back into the ureter. To our experience the most important step is a direct loop in the upper calix to achieve a quick and direct turn back into the ureter. We do acknowledge that there there is a learning curve to the procedure but the steps are easily learned by the residents in our programme. Furthermore there might be anatomical conditions that make this step of the procedure challenging (e.g. a duplex collecting system).

Contrary to our expectations, we did not observe significant differences in operative time between the two groups (67.6 versus 70.3 min, *P =* 0.901). While multiple attempts of directing the guidewire back in the ureter can be time consuming it has been our experience that the actual procedure can be performed more efficiently and possibly faster because of a higher flow of irrigation fluid. This can result in improved vision without an increased risk of stone retropulsation.

This was not a prospective study. Patients were not randomized. By that, the retrospective character and the small number of patients are limitations of this study. Nevertheless, we were able to show the feasibility of this technique and its potential utility in the prevention of stone migration during ureteroscopy and lithotripsy.

## Conclusion

Coiling the routinely used guidewire just proximal to the stone in the ureter prior to lithotripsy during ureteroscopy may be a simple and inexpensive option to significantly reduce inadvertent stone migration and achieve higher stone-free rates.

## Abbreviations

CT: Computerized tomography
fURS: Flexible ureterorenoscopy
Ho:YAG: Holmium:YAG laser
IRB: Institutional review board
IVP: Intravenous pyelography
KUB: Plain film radiographs of the kidneys, ureters, and bladder
SWL: Shock wave lithotripsy
URS: Ureterorenoscopy
